# Widespread occurrence of *Batrachochytrium dendrobatidis* in Ontario, Canada, and predicted habitat suitability for the emerging *Batrachochytrium salamandrivorans*

**DOI:** 10.1002/ece3.8798

**Published:** 2022-04-20

**Authors:** Lauren Crawshaw, Tore Buchanan, Leonard Shirose, Amanda Palahnuk, Hugh Y. Cai, Amanda M. Bennett, Claire M. Jardine, Christina M. Davy

**Affiliations:** ^1^ 6515 Ontario Ministry of Northern Development, Mines, Natural Resources and Forestry Wildlife Research and Monitoring Section Trent University Peterborough ON Canada; ^2^ 3653 Canadian Wildlife Health Cooperative Department of Pathobiology University of Guelph Guelph ON Canada; ^3^ 3653 Department of Pathobiology University of Guelph Guelph ON Canada; ^4^ 3653 Animal Health Laboratory University of Guelph Guelph ON Canada; ^5^ 6515 Department of Biology Trent University Peterborough ON Canada

**Keywords:** amphibian conservation, chytridiomycosis, emerging infectious diseases, fungal pathogens, niche modeling, species distribution modeling

## Abstract

Chytridiomycosis, caused by the fungi *Batrachochytrium dendrobatidis* and *Batrachochytrium salamandrivorans*, is associated with massive amphibian mortality events worldwide and with some species’ extinctions. Previous ecological niche models suggest that *B*. *dendrobatidis* is not well‐suited to northern, temperate climates, but these predictions have often relied on datasets in which northern latitudes are underrepresented. Recent northern detections of *B*. *dendrobatidis* suggest that these models may have underestimated the suitability of higher latitudes for this fungus. We used qPCR to test for *B*. *dendrobatidis* in 1,041 non‐invasive epithelial swab samples from 18 species of amphibians collected across 735,345 km^2^ in Ontario and Akimiski Island (Nunavut), Canada. We detected the pathogen in 113 samples (10.9%) from 11 species. Only one specimen exhibited potential clinical signs of disease. We used these data to produce six Species Distribution Models of *B*. *dendrobatidis*, which classified half of the study area as potential habitat for the fungus. We also tested each sample for *B*. *salamandrivorans*, an emerging pathogen that is causing alarming declines in European salamanders, but is not yet detected in North America. We did not detect *B*. *salamandrivorans* in any of the samples, providing a baseline for future surveillance. We assessed the potential risk of future introduction by comparing salamander richness to temperature‐dependent mortality, predicted by a previous exposure study. Areas with the highest species diversity and predicted mortality risk extended 60,530 km^2^ across southern Ontario, highlighting the potential threat *B*. *salamandrivorans* poses to northern Nearctic amphibians. Preventing initial introduction will require coordinated, transboundary regulation of trade in amphibians (including frogs that can carry and disperse *B*. *salamandrivorans*), and surveillance of the pathways of introduction (e.g., water and wildlife). Our results can inform surveillance for both pathogens and efforts to mitigate the spread of chytridiomycosis through wild populations.

## Graphical abstract

We conducted surveillance for two chytrid fungi (*Batrachochytrium dendrobatidis* and *B*. *salamandrivorans*) in south‐central Canada, in an area where previous niche modeling suggests low habitat suitability for *B*. *dendrobatidis*. Sampling of 1,041 amphibians across Ontario and Akimiski Island (Nunavut) revealed that *B*. *dendrobatidis* is ubiquitous in our study area, but did not detect *B*. *salamandrivorans*. Niche models informed by these data show high habitat suitability for *B*. *dendrobatidis*, and temperature models suggest high habitat suitability for *B*. *salamandrivorans* if it is introduced, emphasizing the need for enhanced coordinated, transboundary regulation of trade and surveillance of potential pathways of transmission.

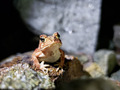

## 1 INTRODUCTION

Increased prevalence of fungal pathogens threatens global biodiversity, agriculture, and human health (Fisher et al., 2012; Lötters et al., 2010). The pathogenic fungi that cause chytridiomycosis (*Batrachochytrium dendrobatidis (Bd)* and *B*. *salamandrivorans (Bsal)*) are a well‐known example of this trend. *Bd* affects a wide range of amphibians and is associated with population declines and even extinction of some species (Lötters et al., 2010). The origins of *Bd* are enigmatic (Rosenblum et al., 2013). Southern Africa and eastern North America were initially proposed as the potential sources of this pathogen (Fisher et al., 2009; Ouellet et al., 2005), but more recent evidence suggests a common ancestral lineage in East Asia (Fisher & Garner, 2020; O’Hanlon et al., 2018). *Bd* now has an almost global distribution and is treated as a potentially invasive species across its contemporary range.

The related, recently discovered “salamander chytrid” *Bsal* is endemic to south‐east Asia but recently spread outside its native range. *Bsal* was introduced to western Europe prior to 2004 through the pet trade (Lötters et al., 2020), and it is associated with rapid population declines of fire salamanders (*Salamandra salamandra*) in the Netherlands and Germany (Lötters et al., 2020; Martel et al., 2013; Spitzen‐van der Sluijs et al., 2013). Populations that survived this devastating outbreak showed no subsequent resistance to *Bsal* (Stegen et al., 2017), suggesting that evolutionary rescue and effective prophylactic treatments (i.e., development of vaccines) are both unlikely. Further spread of *Bsal* is cause for concern because thousands of urodele species endemic to other regions are highly susceptible to this pathogen and would likely experience population declines if it were introduced into their ranges (Martel et al., 2014; Stegen et al., 2017). Some anuran species can also act as reservoir hosts for *Bsal*, potentially increasing the rate of spread following new introductions (Lötters et al., 2020; Nguyen et al., 2017). Understanding and limiting pathogen dispersal are major objectives in the management of emerging wildlife pathogens (Biek & Real, 2010; Wilder et al., 2015). Based on the current evidence, preventing new introductions and slowing range expansion following introductions should be considered the only feasible control measures for *Bsal* (Stegen et al., 2017).

Species distribution models (SDMs) are a common tool used to relate environmental conditions to occurrence data for a species to predict its distribution (Elith & Leathwick, 2009). In the context of pathogen management, SDMs can identify areas to prioritize for surveillance or to limit range expansion (Raffini et al., 2020). SDMs were used to predict the distribution of the “fundamental niche” of *Bd* in the New World and Australia (Rödder et al., 2008; Ron, 2005) and the fundamental niche of *Bsal* in Europe (Feldmeier et al., 2016; Lötters et al., 2020). SDMs have been used to forecast the distribution of *Bd* at a global scale, to quantify associated *Bd*‐related extinction risks for amphibians under a range of climate scenarios, and to predict suitable habitat for *Bsal* in Mexico should an introduction occur (Basanta et al., 2019; Lötters et al., 2010; Olson et al., 2021; Rödder et al., 2009, 2010).

Building an SDM for an invasive species is challenging in the early stages of the invasion when data are limited (Bertolino et al., 2020; Katz & Zellmer, 2018), or when predicting suitable habitat based on occurrence data from regions with different environmental conditions. Field sampling and SDMs have been used to characterize the niche used by *Bd* in tropical regions (Bacigalupe et al., 2019; Catenazzi et al., 2011; Fisher et al., 2009; Kriger & Hero, 2007; Saenz et al., 2015). Models of habitat suitability for *Bd* in Nearctic areas have relied heavily on these data from warmer climates and have predicted low habitat suitability for *Bd* in northern Nearctic regions (Rödder et al., 2010; Xie et al., 2016). However, sampling in Canada detected *Bd* at high latitudes: 60°18′N (Schock et al., 2010); 54°73′N (D’Aoust‐Messier et al., 2015); 46°81′ N (Ouellet et al., 2005). These detections could indicate a recent range expansion—but *Bd* was also detected in museum specimens from Quebec, Canada, collected as early as 1961 (Ouellet et al., 2005). Further sampling across Canada continues to detect *Bd* even with low sampling effort, and reported prevalence in samples from wild populations of Canadian amphibians ranges from 26.9% to 100% (Brunet et al., 2020; D’Aoust‐Messier et al., 2015; Forzán et al., 2010; Jongsma et al., 2019; McMillan et al., 2020; Schock et al., 2010). Taken together, these studies contradict the predictions of available SDMs and suggest *Bd* is established and enzootic in Canada, highlighting the importance of representative sampling to inform SDMs.

Although *Bsal* has not yet been detected in North America, suitable climatic conditions and brisk international trade in amphibians place the continent at high risk of a *Bsal* invasion (Govindarajulu et al., 2017; Waddle et al., 2020; Yap et al., 2017). Identifying potential high‐risk areas now can enable faster responses to slow pathogen spread in the case of an introduction. Basanta et al. (2019) identified high‐risk areas for *Bsal* introduction to Mexico based on environmental conditions and species richness of susceptible salamanders, and a similar approach in Canada could inform surveillance efforts and disease mitigation if necessary.

Northern populations of urodeles may be particularly vulnerable to *Bsal* because its growth and virulence appear to be correlated with temperature. A SDM of *Bsal* in Europe found the fungus can thrive in cool regions and suggested that amphibians in cooler climates may be at higher risk because their immune defenses are largely temperature‐dependent (Feldmeier et al., 2016). This hypothesis was supported by experimental infection of the Nearctic eastern newt (*Notophthalmus viridescens*) with *Bsal*, which led to higher mortality at lower temperatures (Carter et al., 2021). These results shifted concern about *Bsal* northwards from previous risk assessments, identifying the northeastern United States and southeastern Canada as high‐risk areas.

In this study, we aimed to reduce the uncertainty around the drivers and distribution of chytridiomycosis in northern climates. We used SDMs based on local occurrence data to estimate the likely distribution of *Bd* in our study area (Ontario, Canada, and an island of Nunavut just off the coast of Ontario), and we used available data on suitable habitat for *Bsal* to identify particularly high‐risk areas in the region that could be prioritized for surveillance in the case of an introduction. We conducted surveillance for *Bd* and *Bsal* in the study area across a latitudinal gradient of 15 decimal degrees that exceeds the northern range limits predicted by recent SDMs (Rödder et al., 2010; Xie et al., 2016), but within which *Bd* has been shown to occur (D’Aoust‐Messier et al., 2015; McMillan et al., 2020). Our results are intended to inform surveillance activities and mitigation strategies to control these pathogens and their effects on amphibian diversity in temperate climates.

## 2 MATERIALS AND METHODS

### 2.1 Surveillance for *Bd* and *Bsal*

To obtain samples across a wide geographic area, we provided sampling kits to a broad network of government biologists and interested researchers, who opportunistically swabbed any amphibians they encountered during their work across the province of Ontario and on Akimiski Island, Nunavut (Canada). Sampling methods were approved by the Wildlife Animal Care Committee of the Government of Ontario and by Ontario Parks. Briefly, participants identified each amphibian they captured to species. They recorded potential clinical signs of chytridiomycosis, but collected swab samples from each individual regardless of clinical signs. Participants wore new nitrile gloves to handle each individual to avoid cross‐contamination of samples. Samples were collected by rubbing sterile swabs (Puritan, 6” Sterile Polyester Tipped Applicator 25‐806 1PD) on the ventral surface, under the forelimbs, and along the sides of the mouth. Tadpole swabbing focused on the mouth area where the fungus is able to grow on the keratinized mouthparts. Swabs were rotated while sampling to ensure all sides of the swab made contact. Samples were stored in lysis buffer, kept cool and out of direct sunlight while in the field, and then stored in fridges and/or freezers (as available) prior to laboratory analysis at the Animal Health Laboratory (AHL) in Guelph, Ontario. We used qPCR analysis of the samples to estimate distribution and prevalence of *Bd* and to conduct surveillance for *Bsal*.

The AHL facility follows strict quality control procedures, including use of a separate DNA extraction room, master mix assembly room, and amplification room. Positive control and negative control samples were included in all procedures from DNA extraction to amplification. We extracted DNA from the swab samples using a commercial DNA extraction kit (DNeasy Plant Mini Kit, Qiagen, Toronto, Ontario) and following the manufacturer's protocols. Real‐time polymerase chain reaction (qPCR) assays were performed to detect *Bd* and *Bsal*, as described previously (Blooi et al., 2013, 2016), and the qPCR assays conducted at the AHL are ISO/IEC 17025 accredited and periodically validated to ensure accuracy. To avoid cross‐talk between the Cy5 and Fam fluorescent markers, we performed the duplex qPCR as two simplex qPCRs. Samples were run singly, but we confirmed inconclusive (borderline positive) samples by retesting in triplicate. We scored samples with cycle threshold (CT) values (CT) >40 as negative for the target (*Bd* or *Bsal*). This conservative approach reduces the risk of false positives, but may underestimate the true prevalence of the target pathogens. Thus, our data represent an estimate of minimum prevalence.

We tested for spatial autocorrelation among sampling locations using the join count test from the R package *spdep* (Bivand & Wong, 2018).

### 2.2 Exploring the distribution of *Batrachochytrium dendrobatidis*

#### 2.2.1 Latitudinal distribution of *Bsal* detection

To explore the hypothesis of a northern range limit for *Bd*, we tested the association between *Bd* prevalence and sampling latitude using logistic regression in R 4.0.3 (R Core Team, 2020). We restricted this analysis to those species with a sample size >100 that were well‐sampled across a broad latitudinal range (*Lithobates sylvaticus*, *L*. *clamitans*, *L*. *pipiens*, and *Anaxyrus americanus*).

#### 2.2.2 Species distribution models—*Batrachochytrium dendrobatidis*

We created six SDMs in Maxent version 3.4.1 (Phillips et al., 2006) to identify suitable habitat for *Bd* across our study area using our detected cases and 20 environmental variables. Maxent is an open‐source program that uses presence points and a maximum entropy algorithm to estimate the probability of species occurrence when constrained by environmental conditions. Maxent has high discriminatory power (Bradie & Leung, 2017) and has been used previously to create SDMs for *Bd* and *Bsal* (Bacigalupe et al., 2019; Basanta et al., 2019; Lötters et al., 2010; Rahman, 2020; Rödder et al., 2008; Ron, 2005; Seimon et al., 2015). These studies used 19 bioclimatic variables from WorldClim 2.1 (https://worldclim.org/data/index.html) (Fick & Hijmans, 2017) (Table 1) and found that many are predictors of *Bd* habitat suitability and chytridiomycosis severity. We also used these variables, but instead of using the 50‐year averages from WorldClim, we recreated these variables for the period of our study (2014–2017) using data from the Daily Surface Weather Data version 3 (Daymet). This NASA‐sourced raster dataset includes monthly summaries of daily weather parameters across North America at a 1 km resolution (Thornton et al., 2017). We downloaded 4 years of temperature and precipitation data and used the R package *dismo* to recreate the 19 bioclimatic variables for each year (Hijmans et al., 2021). After averaging each variable across years, they were checked for errors using reference code available through the USGS (O’Donnel & Ignizio, 2012). To explore the effect of elevation on the distribution of *Bd*, we also included a Digital Elevation Model (DEM) from the Shuttle Radar Topography Mission (NASA JPL, 2013). We resampled this DEM to a 1 km grid to match the bioclimatic variables. We initially considered also including an urbanization variable, distance to roads, to account for potential human impacts on *Bd* occurrence. However, almost all of our samples were collected in close proximity to roads due to our opportunistic sampling design. Thus, this variable could have simply confounded the models (i.e., resulted in predictions that sites far from roads were not suitable habitat simply because our sampling was biased toward sites near roads), and we did not include this variable in our analyses.

**TABLE 1:** Environmental variables used in the Maxent models (NASA JPL, 2013)

Variable	*Bd* Models		*Bsal* Models	
	*Full*	*Reduced*	*Full*	*Reduced*
Bioclimatic variables				
BIO1—Annual Mean Temperature	x		x	
BIO2—Mean Diurnal Range	x	x	x	
BIO3—Isothermality (= BIO2/BIO7)	x	x	x	x
BIO4—Temperature Seasonality	x		x	
BIO5—Max Temperature of Warmest Month	x	x	x	x
BIO6—Min Temperature of Coldest Month	x	x	x	
BIO7—Temperature Annual Range	x		x	x
BIO8—Mean Temperature of Wettest Quarter	x	x	x	
BIO9—Mean Temperature of Driest Quarter	x		x	
BIO10—Mean Temperature of Warmest Quarter	x		x	
BIO11—Mean Temperature of Coldest Quarter	x		x	
BIO12—Annual Precipitation	x		x	
BIO13—Precipitation of Wettest Month	x		x	
BIO14—Precipitation of Driest Month	x		x	
BIO15—Precipitation Seasonality	x	x	x	x
BIO16—Precipitation of Wettest Quarter	x	x	x	x
BIO17—Precipitation of Driest Quarter	x		x	x
BIO18—Precipitation of Warmest Quarter	x	x	x	
BIO19—Precipitation of Coldest Quarter	x		x	
Digital Elevation Model	x	x		

Bioclimatic variables for the *Bd* and *Bsal* models were sourced from Daymet (Thornton et al., 2017) and WorldClim 2.1 (Fick & Hijmans, 2017), respectively.

Our six models were constructed using a combination of variable sets (Full set vs Reduced set based on a correlation analysis), and three different bias grids (a raster surface that represents sampling effort, cropped to a buffer of 0.5, 1, and 2 decimal degrees) that would restrict background point selection to address sampling bias. A Maxent model's goodness of fit is estimated by gain (Phillips et al., 2017), which can be increased by addressing collinearity and reducing the environmental layer inputs in the model (Kramer‐Schadt et al., 2013). We created a correlation matrix of variables listed in Table 1 to identify highly correlated variables (Table A1; *R* > .75). We ran logistic regression among these and retained the variable that showed the strongest association with *Bd* presence based on point‐wise values from all 1,041 sample locations. In cases where the difference was small and the choice was between variable representing averages and weather extremes, we retained the extremes as these are most relevant to niche limits (Lötters et al., 2010; Seimon et al., 2015). This process identified the nine variables used in the “Reduced” models (columns 1 and 2, Table 1).

Maxent is robust when handling multicollinearity among predictor variables, but there is uncertainty on whether to include all potential variables into an SDM or to reduce the number of variables *a priori* (Feng et al., 2019). In ambiguous situations where the drivers of species and climate relationships are unknown, retaining one variable from a highly correlated cluster may cause the future range predictions to vary considerably (Braunisch et al., 2013). Therefore, we also ran full models containing all 20 variables for each of the three bias grids. Our goal was not to identify a single, best‐fit model, but to evaluate the qualitative agreement among biologically informative model outputs (i.e., how robust were our results to different model parameters).

We converted all 20 Daymet variables listed in Table 1 to the same coordinate system (WGS 1984) before clipping them to a 5 km‐buffered boundary of the sampling area, which included the province of Ontario and Akimiski Island (Nunavut) for processing in Maxent version 3.4.1 (Phillips et al., 2006). Maxent analyses rely on the assumption that detection probability is constant across all sites (Phillips et al., 2009), although this is frequently violated in practice (Yackulic et al., 2013). Uneven sampling effort can be addressed by thinning the number of occurrences (spatial filtering), by manipulating the background data to account oversampled areas (i.e., using a bias grid), and by reducing multicollinearity (Kramer‐Schadt et al., 2013). Most of the samples were collected in southern Ontario, while the remote north‐west region of the province was sparsely sampled. We applied a spatial filter to the data by retaining only one positive case point per km^2^ (Kramer‐Schadt et al., 2013) and used bias grids to correct for the possible effects of the sampling distribution on the model output (Fourcade et al., 2014). These grids were created using the Gaussian Kernel density of sampling localities tool in the SDM Toolbox v2.4 in ArcGIS 10.3 (Brown et al., 2017). As there is no defined rule for selecting background sampling localities, we used three different sampling bias thresholds (0.5, 1, and 2 decimal degrees). These were clipped to the same extent as the other rasters and inputted into Maxent as a bias layer.

For all six models, the optimal model settings were first selected using the R package *ENMeval* prior to running in Maxent. This package's function *ENMevaluate* helps fine‐tune ecological niche models by running multiple iterations of user defined specifications for the regularization multiplier (RM) and Maxent feature types (e.g., linear) (Muscarella et al., 2014). The RM is a smoothing parameter that adds constraints to the model to prevent overfitting (Phillips & Dudík, 2008). We used RM increments of 0.5, between 0.5 and 2, with four combinations of linear, quadratic, product, and hinge features (“L,” “LQ,” “LQP,” and “LQH”) to create 20 candidate models using the random‐fold method and 10 k‐folds. We selected the models with the lowest delta Akaike's information criterion for small sample sizes (ΔAICc), a measure of goodness of fit that penalizes overparameterization (Muscarella et al., 2014). The best‐supported model determined the RM and feature settings in Maxent.

We used the default ClogLog output format in Maxent, which provides an estimate of probability of presence between zero and one for each pixel. It is considered the most appropriate for estimating probability of presence, although it often estimates higher suitability values than the logistic output (Phillips et al., 2017). We partitioned *Bd* occurrence data into two groups (75% training, 25% testing) and tested the Maxent models with cross‐validation using the default of a maximum of 10,000 randomly sampled background points. We set the maximum number of iterations to 5,000 and ran 10 replicates of each model, using the averaged suitability probabilities for our analyses.

To evaluate the individual model performance, we used receiver operating characteristic (ROC) area under the curve (AUC). A model with an AUC = 0.5 has no discrimination ability, while models with an AUC > 0.7 have adequate power of discrimination (Hosmer et al., 2013). We further evaluated the predictive accuracy of our models by calculating the true skill statistic (TSS) (Sensitivity + Specificity − 1; please see Allouche et al., 2006 for details). Values range between 0 and 1, with higher values indicating better goodness of fit. We took the average TSS for the 10 replicate runs. We evaluated the relative importance of each predictor variable using Maxent's Jackknife tests, which show the changes in gain when each variable is either removed from model or is the only variable retained.

The 10th percentile minimum training presence ClogLog threshold was used to create binary maps of habitat suitability for the study area. This threshold omits records with habitat suitability <10 percent during model training based on the assumption that the outlying locations do not represent typical habitat. This threshold performed the best in a previous SDM used to identify areas of endemism in North American mammals (Escalante et al., 2013) and is less sensitive to extreme localities (Radosavljevic & Anderson, 2014). AUC does not quantify overfitting and may result in overly complex models. Therefore, comparisons of omission rates from Maxent's output were used to assess overfitting in each model, which affects the model's generality, by comparing the observed values to the expected result.

### 2.3 Predicting the distribution of *Batrachochytrium salamandrivorans*

We used two approaches to identify likely high‐risk areas for the introduction of *Bsal* in our study area. First, we replicated the approach used by Basanta et al. (2019) in Mexico to predict likely suitable habitat for *Bsal* within our study area, based on the environmental conditions in areas where *Bsal* is native or has become established. Second, we mapped the ranges of the species in our study area that are likely to be susceptible to *Bsal*, identified areas of high species richness based on those ranges, and overlaid climate variables associated with severe *Bsal* chytridiomycosis (Carter et al., 2021) to identify high‐priority areas for surveillance in case of an introduction.

#### 2.3.1 Species distribution models—*Batrachochytrium salamandrivorans*

Basanta et al. (2019) used *Bsal* presence data from Asia and Europe to predict where *Bsal* might thrive in Mexico if introduced and then compared the output to species richness in the region to identify hotspots of conservation concern. We replicated this approach for our study area using 44 *Bsal* occurrence records summarized in Basanta et al. (2019) and 171 additional records from Lötters et al. (2020), sourced from Beukema et al. (2018), Dalbeck et al. (2018), Laking et al. (2017), Lötters et al. (2018), Martel et al. (2014), Schulz et al. (2018), Spitzen‐van der Sluijs et al. (2016), Wagner et al. (2019), and Yuan et al. (2018). Daymet is unavailable outside North America, so we used the 19 bioclimatic variables from WorldClim 2.1 to obtain comparable environmental data for the current *Bsal* range and our study area (Fick & Hijmans, 2017). We selected terrestrial ecoregion boundaries from https://databasin.org that overlapped with *Bsal* cases to clip the raster layers in Asia and Europe (Olson et al., 2001). We selected bioclimatic variables previously associated with *Bsal* prevalence (Table 2), and these eleven bioclimatic rasters were tested for multicollinearity (*R* > .75) as described above for the *Bd* models. The only difference was the use of 100 randomly sampled pseudoabsences to compare to the *Bsal* positives in the logistic regression tests.

**TABLE 2:** Variables found to have strong association with *Bsal* prevalence in previous literature

Source	Variable related to *BSAL* prevalence
Basanta et al. (2019)	**Minimum temperature of the coldest month (BIO5)** **Temperature annual range (BIO7)** Precipitation seasonality
Martel et al. (2013)	Salamander richness Trade risks (e.g., active ports for importing salamanders) Urodele family
More et al. (2018)	Spread by passive carriers (e.g., water, birds) Human activities, waste water, equipment, fomites Connected waterways
Beukema et al. (2018)	**Isothermality (BIO3)** Temperature seasonality (BIO4) **Maximum temperature of the warmest month (BIO5)** Minimum temperature of the coldest month (BIO6) **Precipitation seasonality (BIO15)** **Precipitation of the wettest quarter (BIO16)** **Precipitation of the driest quarter (BIO17)**
Feldmeier et al. (2016)	Days with minimum temperatures between 10° and 15°C Highest number of consecutive days warmer than 25°C Mean temperature of the coldest quarter (BIO11) **Precipitation of the driest quarter (BIO17)**
Katz and Zellmer (2018)	Salamander richness Number of salamander imports **Isothermality (BIO3)** Temperature seasonality (BIO4) Mean temperature of the warmest quarter (BIO10) Precipitation of the wettest month (BIO13) Precipitation of the driest month (BIO14)
Yap et al. (2017)	Temperatures 5–25°C, with optimal growth at 10–15°C *Bsal* was detected on salamanders in ponds where water temperatures were over 26°C

The six bioclimatic variables in bold were selected through correlation analysis and used to predict habitat suitability for *Bsal* in the present study.

To predict habitat suitability for *Bsal* in our study area, we constructed six SDMs based on the Asian and European occurrence data, spatially filtered so that each square kilometer only contained one occurrence. Two SDMs used all 215 cases, one with the full set of predictor variables and one using the reduced set. We also ran full and reduced models using only the occurrence data from Asia (*N* = 30) and only that from Europe (*N* = 185). We did not include elevation in these models because of the large difference in elevation between Ontario and the native range of *Bsal* (>3,400 m).

The R function *ENMevaluate* was used again to optimize the settings for each model, with RM values set to increments of 0.5 between 0.5 and 2.5, with four possible feature classes (L, LQ, LQP, LQH). We used the random‐fold method for the two models with the complete dataset, and the jackknife method for the Asia‐only and Europe‐only models because of their smaller sample size (Muscarella et al., 2014). These outputs provided the optimized settings for the *Bsal* models in Maxent, which were set to 5000 iterations. A 100 km bias grid buffer around the occurrences was used to restrict the selection of 10,000 background points. Finally, we reclassified the ClogLog output of the projected suitability maps for *Bsal* in Ontario from high (>0.75) to no suitability (<25).

### 2.4 Predicting high‐risk areas for *Bsal* infection

We aggregated distribution data for *Bsal*‐susceptible salamander species to quantify the distribution of species at high risk from *Bsal* in our study area. These included Salamandrid, Proteid, and Plethodontid species that are thought to be vulnerable to *Bsal* infections (Carter et al., 2021; Martel et al., 2014; O’Hanlon et al., 2018). Genomic analysis and laboratory tests suggest that Ambystomatid salamanders are resistant to *Bsal* infection (Barnhart et al., 2020; Martel et al., 2014; Pereira & Woodley, 2021) (Table A2), although they may still be able to carry and spread *Bsal*. Our objective was to identify priority areas for future surveillance for *Bsal* in the ranges of vulnerable species, so *Ambystoma* was excluded from this prioritization analysis.

We included occurrence records for vulnerable species from our study results (*N* = 97) and from occurrence data archived in the Global Biodiversity Information Facility since 1950 (GBIF, 2020; *N* = 4410). The final dataset included *Notophthalmus viridescens* (*N* = 1120), *Necturus maculosus* (*N* = 234), *Plethodon cinereus* (*N* = 2843), *Eurycea bislineata* (*N* = 193), and *Hemidactylium scutatum* (*N* = 117). *Desmognathus fuscus* and *D*. *ochrophaeus* occur in Ontario, but were excluded due to insufficient data. We used these occurrence data to redraw range maps for each species, modifying the range maps produced by International Union for Conservation of Nature (Hammerson, 2004; IUCN SSC ASG, 2015a, 2015b, 2015c, 2015d). The final range maps for each species were then overlayed using the Union tool in R to create a map of species richness.

Carter et al. (2021) used trials of *Bsal* infection severity at different temperatures to map the risk of *Bsal* invasion to *N*. *viridescens*, the eastern newt. In these laboratory tests, adult and juvenile *N*. *viridescens* died sooner from *Bsal* infection at 14°C than at 6 or 22°C, with 90 percent mortality at 6°C and 14°C, though animals died 1.4 times slower at 6°C. Carter et al. (2021) used these thresholds to reclassify temperature variables and predict *Bsal* mortality risk across the range of *N*. *viridescens*. In the absence of available data for other species, we made an explicit assumption that these thresholds can be applied to other susceptible species. We replicated the approach of Carter et al. (2021) in our study area using average annual temperature and maximum temperature of the warmest month variables (BIO1 and BIO5 from Daymet, Table 1). For each variable, temperatures between 6 to 14°C were assigned the highest risk to *Bsal* (risk score = 4), while temperatures 22°C or greater classified as lowest risk (risk score = 1). All temperatures below 6°C were scaled from 1 to 4, and temperatures between 14°C and 22°C scaled from 4 and 1 (Carter et al., 2021). After averaging the two layers, we overlapped the result with the salamander species richness map to identify areas of high *Bsal* risk to native salamanders.

## 3 RESULTS

### 3.1 Surveillance for *Bd* and *Bsal*

**FIGURE 1:**
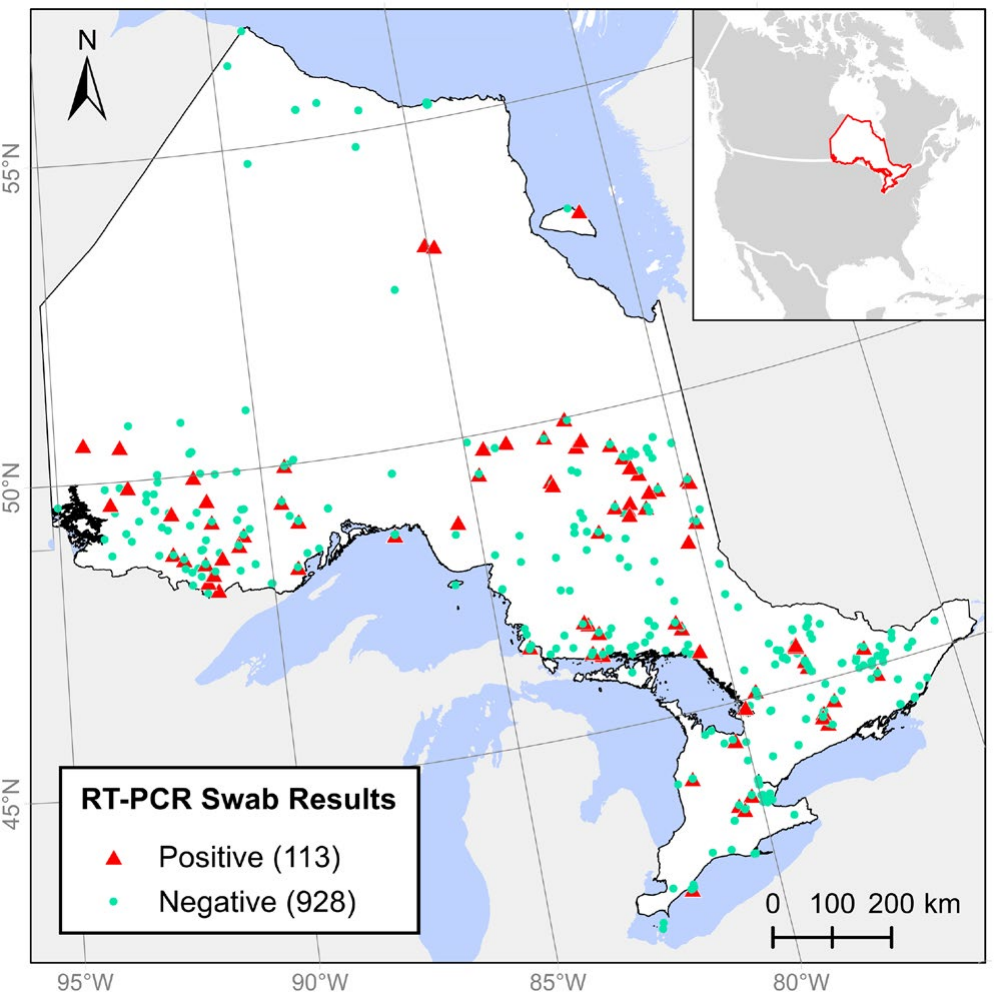
Surveillance for *Bd* and *Bsal* from 2014–2017. 1041 animals were tested across 18 species in Ontario and Akimiski Island in James Bay. 113 tested positive for *Bd* (10.9%). None tested positive for *Bsal*

**FIGURE 2:**
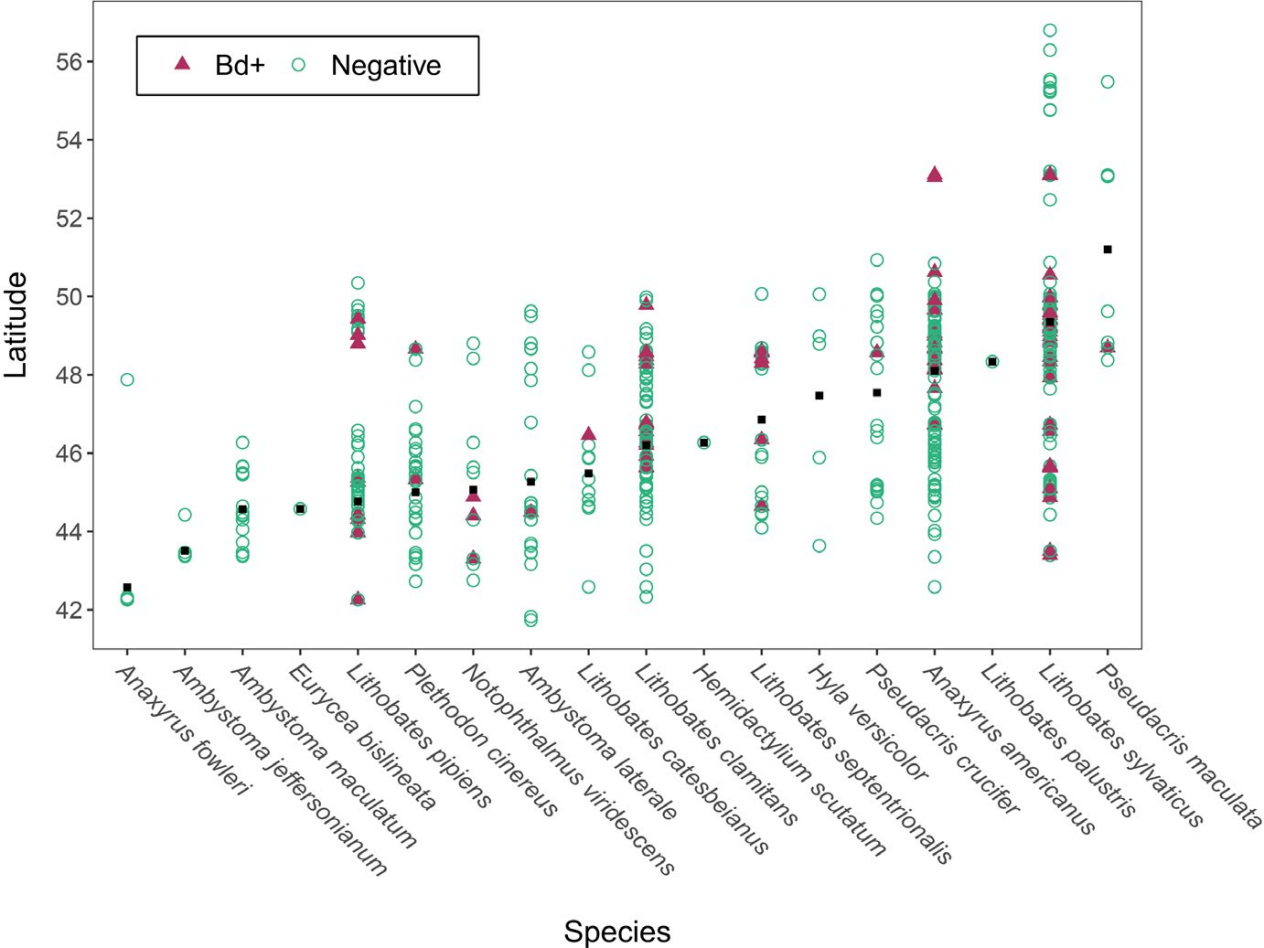
Distribution of *Bd* results with qPCR by latitude and species. The black squares represent average latitude for each species

We collected 1,041 skin‐swab samples from 18 species of frogs and salamanders across 735,345 km^2^ and a latitudinal gradient of 15.07 decimal degrees (41°73′N–56°80′N), and tested these for *Bd* and *Bsal* using qPCR (Figures 1 and 2; Table 3). We detected *Bd* in 113/1041 samples (10.9%) that were distributed across our study area, with the exception of the far north of Ontario where our sampling was limited. Ten samples from 2015 produced inconclusive results for *Bd* (clear amplification curve but CT > 40), and we scored these as negative. We did not detect *Bsal* in any of the samples.

**TABLE 3:** Detection of *Bd* by qPCR from epithelial swab samples (total swabs (*Bd*+swabs)) collected across the study area shown for each family (in bold) and species (in italics)

Species	Samples (Total (*Bd*+)			Prevalence of *Bd*					Combined sample	Combined prevalence
	2014	2015	2016	2017	2014	2015	2016	2017		
Frogs and toads (ANURA)										
True frogs (Ranidae)	**131 (27)**	**348 (37)**	**109 (11)**	**29 (8)**	**0.21**	**0.11**	**0.10**	**0.28**	**617 (83)**	**0.13**
*Lithobates sylvaticus*	87 (16)	107 (17)	46 (4)	23 (7)	0.18	0.16	0.09	0.30	263 (44)	0.17
*Lithobates clamitans*	35 (8)	128 (10)	7 (0)		0.23	0.08	0.00		170 (18)	0.11
*Lithobates pipiens*	2 (0)	107 (9)	10 (2)	1 (1)	0.00	0.08	0.20	1.00	120 (12)	0.10
*Lithobates septentrionalis*	5 (3)	5 (1)	29 (4)	5 (0)	0.60	0.20	0.14	0.00	44 (8)	0.18
*Lithobates catesbeianus*	2 (0)	1 (0)	16 (1)		0.00	0.00	0.06		19 (1)	0.05
*Lithobates palustris*			1 (0)				0.00		1 (0)	0.00
Tree Frogs (Hylidae)	**2 (0)**	**21 (1)**	**13 (1)**	**8 (0)**	**0.00**	**0.05**	**0.08**	**0.00**	**44 (2)**	**0.05**
*Pseudacris crucifer*		17 (1)	6 (0)	6 (0)		0.06	0.00	0.00	29 (1)	0.03
*Pseudacris maculata*	1 (0)	4 (0)	5 (1)		0.00	0.00	0.20		10 (1)	0.10
*Hyla versicolor*	1 (0)		2 (0)	2 (0)	0.00		0.00	0.00	5 (0)	0.00
Toad*s* (Bufonidae)	**30 (7)**	**68 (5)**	**84 (7)**	**16 (3)**	**0.23**	**0.07**	**0.08**	**0.19**	**198 (22)**	**0.11**
*Anaxyrus americanus*	30 (7)	65 (5)	68 (7)	16 (3)	0.23	0.08	0.10	0.19	179 (22)	0.12
*Anaxyrus fowleri*		3 (0)	16 (0)			0.00	0.00		19 (0)	0.00
Salamanders (CAUDATA)										
Mole Salamanders (Ambystomatidae)		**62 (1)**	**20 (0)**	**3 (0)**		**0.02**	**0.00**	**0.00**	**85 (1)**	**0.01**
*Ambystoma laterale* ^a^		31 (1)	17 (0)	3 (0)		0.03	0.00	0.00	51 (1)	0.02
*Ambystoma maculatum*		23 (0)	1 (0)			0.00	0.00		24 (0)	0.00
*Ambystoma jeffersonianum*		8 (0)	2 (0)			0.00	0.00		10 (0)	0.00
Lungless Salamanders (Plethodontidae)		**51 (2)**	**34 (0)**			**0.04**	**0.00**		**85 (2)**	**0.02**
*Plethodon cinereus*		50 (2)	33 (0)			0.04	0.00		83 (2)	0.02
*Eurycea bislineata*			1 (0)				0.00		1 (0)	0.00
*Hemidactylium scutatum*		1 (0)				0.00			1 (0)	0.00
Newts (Salamandridae)		**6 (2)**	**6 (1)**			**0.33**	**0.17**		**12 (3)**	**0.25**
*Notophthalmus viridescens*		6 (2)	6 (1)			0.33	0.17		12 (3)	0.25
TOTALS	163 (34)	556 (48)	266 (20)	56 (11)	0.21	0.09	0.08	0.20	1,041 (113)	0.11

Each swab was also tested for *Bsal*, but that pathogen was not detected in any sample.

^a^ Five of these individuals were identified as possible *laterale*‐*jeffersonianum* hybrids.

**FIGURE A1:**
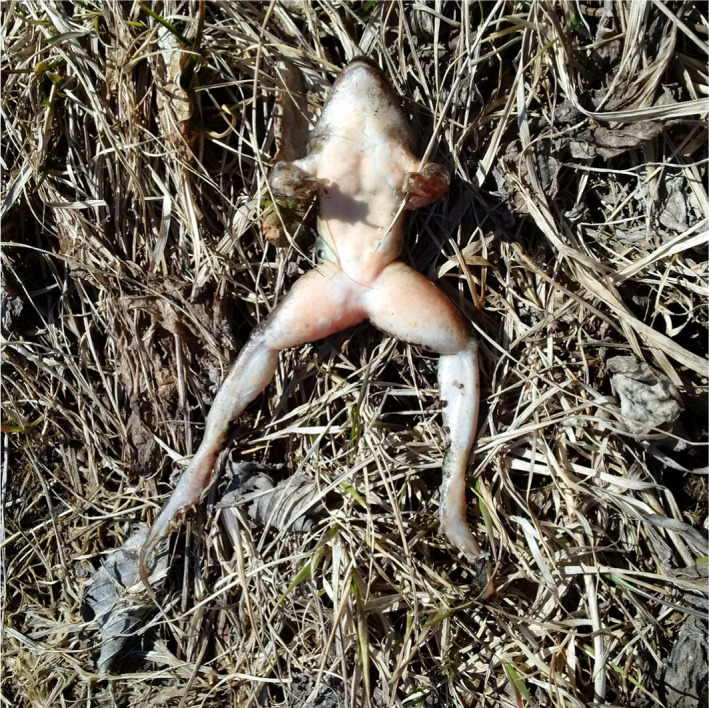
Deceased *Lithobates pipiens* that tested positive for *Batrachochytrium dendrobatidis* near Peterborough, Ontario, Canada

Prevalence of *Bd* varied among species and years, but we detected *Bd* in 11/18 sampled species (eight species of frogs, three species of salamander), and in all species with sample sizes >25. Yearly prevalence in our samples (number of positives/total number of samples per year) from 2014 to 2017 was 20.6%, 8.6%, 7.5%, and 19.6%, respectively, but sampling locations varied geographically each year. Swabbed individuals appeared healthy and did not exhibit obvious clinical signs of disease, with two exceptions: (1) one adult *L*. *sylvaticus* had reddish skin on its legs, but tested negative for *Batrachochytrium* and (2) a mature, deceased *L*. *pipiens* collected near Peterborough, Ontario, tested positive for *Bd* (Figure A1).

The detected prevalence of *Bd* increased as the number of sampled animals increased. This trend was borderline significant using linear regression (*p* = .057), though the sample size was small (i.e., 18 species). Sampling locations were spatially autocorrelated (*p* < .0001), and most were near roads due to the opportunistic nature of the sampling.

### 3.2 Exploring the distribution of *Batrachochytrium dendrobatidis*

#### 3.2.1 Latitudinal distribution

There was no linear association between *Bd* detection and latitude for well‐sampled species (>100 occurrences/species; *N* = 732; *p* = .19), nor any apparent association for the other species (Figure 2).

#### 3.2.2 Species distribution models—*Batrachochytrium dendrobatidis*

**FIGURE 3:**
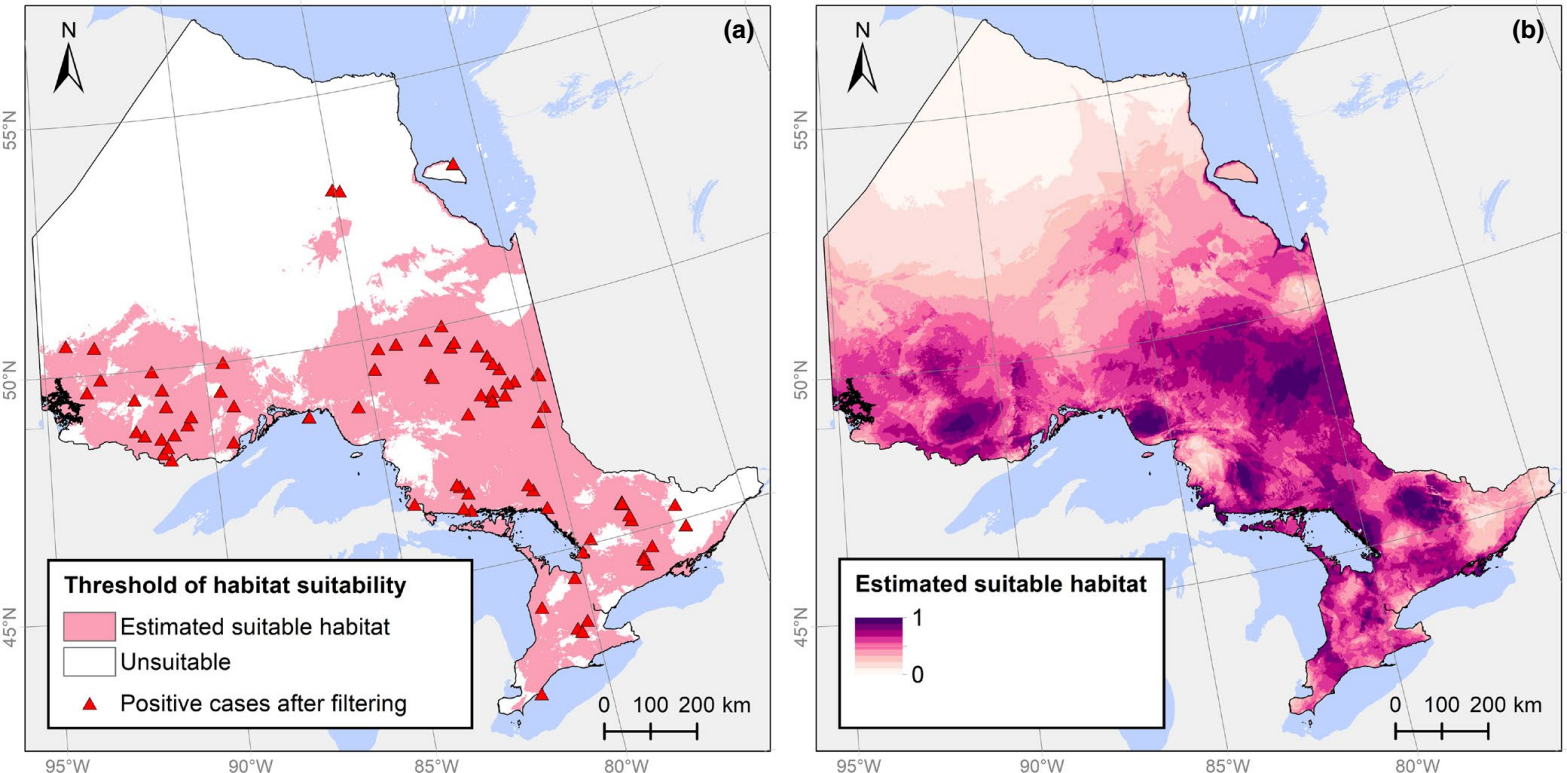
(a) Predicted habitat suitability of *Bd* using the 10th percentile training presence ClogLog threshold from the study area. Solid shading indicates suitable habitat, which are overlayed with the 86 positive spatially filtered occurrence points used to create this Maxent model. (b) Predicted habitat suitability for the best model (Reduced, Bias Grid 0.5). This model was created using nine predictor variables (Table 1)

**FIGURE A2:**
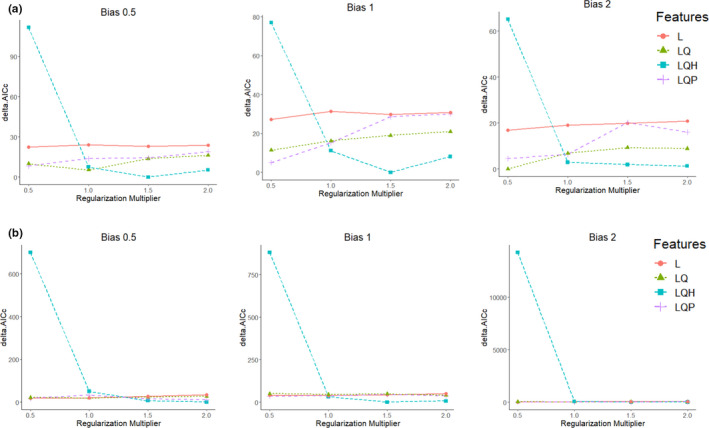
Results of *ENMevaluate* in R for selecting Regularization Multiplier (RM) for the (a) Reduced *Bd* Models and (b) Full *Bd* models will 20 variables. Lower ∆AICc values indicated a better model

We retained 86 of the 113 *Bd* occurrences after spatial filtering (Figure 3a). Results from the six *Bd* SDMs are summarized in Table 4, and all performed best with a combination of linear, quadratic, and hinge features (LQH) with a RM of 1.5 or 2 (Figure A2). All model AUC values were above 0.73 (±0.03) for training and 0.68 (±0.03) for test data, with an average of 0.82 and 0.74, respectively, indicating moderate performance as classifiers. The Full model with the 0.5 Bias Grid (Full 0.5 model) had the highest AUC test score (0.79) followed by the Reduced 0.5 model (0.78).

**TABLE 4:** Summary statistics for the six Maxent suitability models of *Bd*

Model Type	Feature	RM	Training AUC (Average)	Test AUC	AUC Standard Deviation	10 percentile training presence ClogLog threshold	% of study area classified as suitable habitat using ClogLog threshold	% of study area classified as highly suitable habitat (>0.6)	Omission Rate (10th percentile training presence test omission)	Average true skill statistic (TSS)
Reduced model										
Bias 0.5	LQH	1.5	0.85	0.78	0.07	0.50	44.6	30.2	0.18	0.47
Bias 1	LQH	1.5	0.83	0.74	0.08	0.50	53.5	33.0	0.18	0.38
Bias 2	LQ	0.5	0.73	0.68	0.09	0.37	76.2	37.2	0.11	0.16
Full model										
Bias 0.5	LQH	2	0.85	0.79	0.07	0.49	47.6	33.5	0.11	0.45
Bias 1	LQH	1.5	0.84	0.75	0.07	0.50	45.9	28.7	0.20	0.45
Bias 2	LQH	1.5	0.82	0.73	0.08	0.46	48.4	28.4	0.27	0.41
Average			**0.82**	**0.74**	**0.07**	**0.47**	**52.7**	**31.8**	**0.18**	**0.39**

Across the six models, an average of 483,032 km^2^ (52.7%) of the study area was classified as suitable habitat for *Bd* using the 10th percentile minimum training presence ClogLog threshold. The proportion of suitable area ranged from 44.6% to 76.2% and increased with each background extent. Assuming that a threshold >0.6 indicates high suitability (Stabach et al., 2009), 28.4% to 37.2% of the study area was classified as highly suitable in the six models (Table 4). All models had omission rates below 27% (mean omission rate: 18%), and the models with the best omission rates (closest to 10%, the theoretical expectation) were the Reduced 2.0 model and Full 0.5 model, which each had omission rates of 0.11 indicating lower overfitting.

**FIGURE A3:**
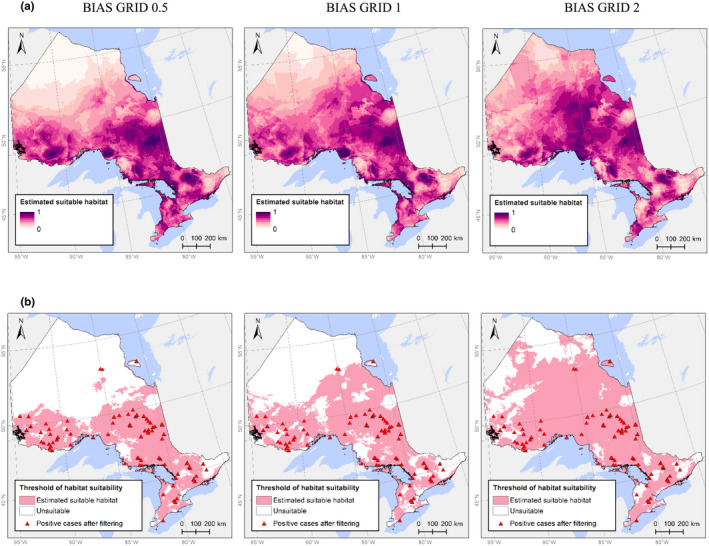
(a) Reduced Model outputs of predicted habitat suitability of *Bd* using the ClogLog distribution using three different background extents for the bias grid (0.5, 1, and 2 decimal degrees). (b) The 10th percentile training presence ClogLog threshold for each background bias grid. Shading indicates the area of habitat suitability. The reduced models were created with nine predictor variables listed in Table 1

**FIGURE A4:**
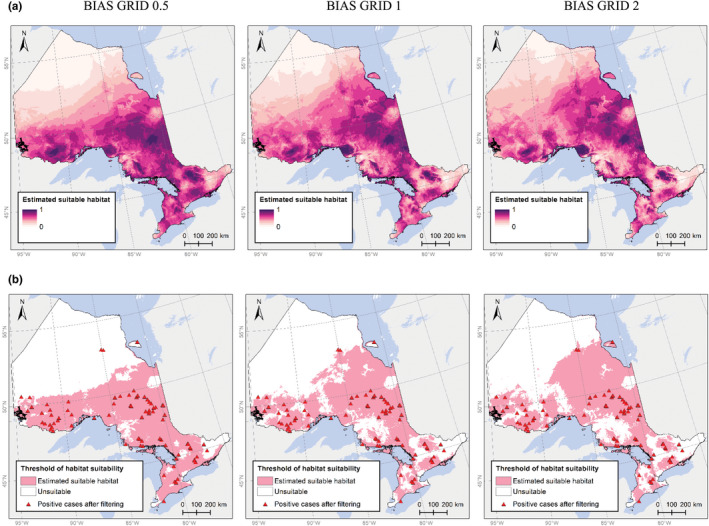
(a) Full Model outputs of predicted habitat suitability of *Bd* using the ClogLog distribution using three different background extents for the bias grid (0.5, 1, and 2 decimal degrees). (b) The 10th percentile training presence ClogLog threshold for each background bias grid. Shading indicates the area of habitat suitability. The full models were created with 21 variables listed in Table 1

The Reduced 0.5 model was selected as the best model due a high AUC score, the highest TSS score (0.47) and the most conservative prediction of *Bd* habitat suitability (44.6%), although the omission rate was moderate (0.18). Figure 3a,b present the mapping output for the Reduced 0.5 model and the area classified as suitable using the 10th percentile threshold, respectively. Maps based on the other five models are available in the Appendix A (Figure A3, Figure A4). Results were similar, indicating that these models were qualitatively robust to variations in model inputs and settings.

**FIGURE A5:**
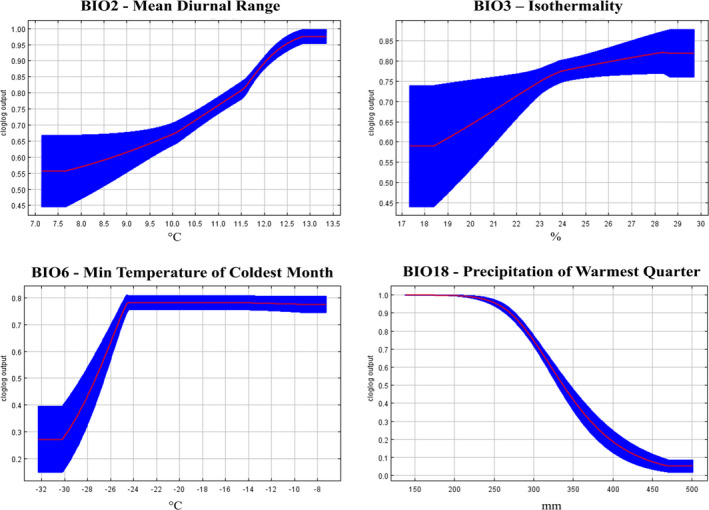
Response curves created in Maxent of the most important variables in the best Maxent model (Reduced 0.5) when controlling for other variables

Three variables contributed to 68.5% of the variability and drove performance of the Reduced 0.5 model (Table A3). Minimum Temperature of the Coldest Month (BIO6) made the greatest contribution (41.2%) to the predicted probability of *Bd* presence. Habitat suitability was positively correlated with BIO6 in places with minimum temperatures below −25°C, above which the response curve flattens out (Figure A5). Mean Diurnal Range (BIO2) was the second greatest contributor (16.7%). As the range in diurnal temperature widened, the probability of *Bd* presence increased. The model‐predicted probability of *Bd* distribution also increased with Isothermality (BIO3; 10.6%), a ratio comparing day to night temperature oscillations to summer/winter oscillations, represented as a percent (O’Donnel & Ignizio, 2012).

**FIGURE A6:**
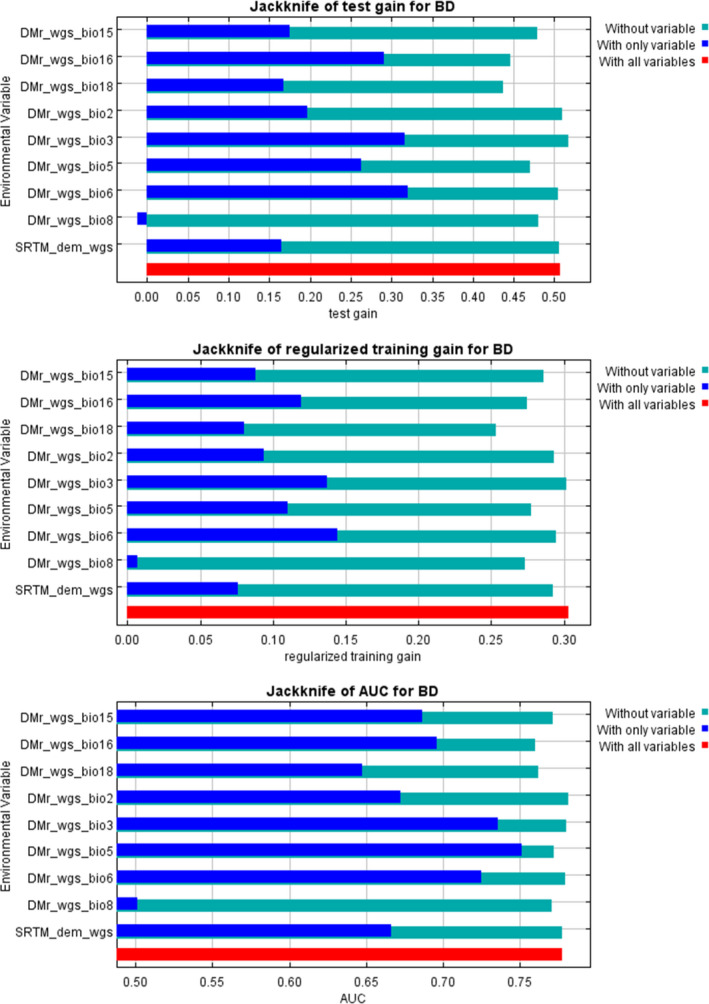
Jackknife tests for evaluating the relative importance of each environmental variable in the Reduced 0.5 model. These raw plots created in Maxent show the change in gain when each variable is removed from the full model (light blue bar), or when it is the only variable included (dark blue bar)

The jackknife test evaluating the relative importance of each environmental variable showed that Minimum Temperature of Coldest Month (BIO6), followed by Isothermality (BIO3), produced the highest gain when these variables were used alone in the model. Removing Precipitation of the Warmest Quarter (BIO18) from the model reduced gain more than exclusion of any other variable, suggesting that BIO18 improves model fit (Figure A6).

### 3.3 Predicting the distribution of *Batrachochytrium salamandrivorans*

#### 3.3.1 Species distribution models – *Batrachochytrium salamandrivorans*

**FIGURE A7:**
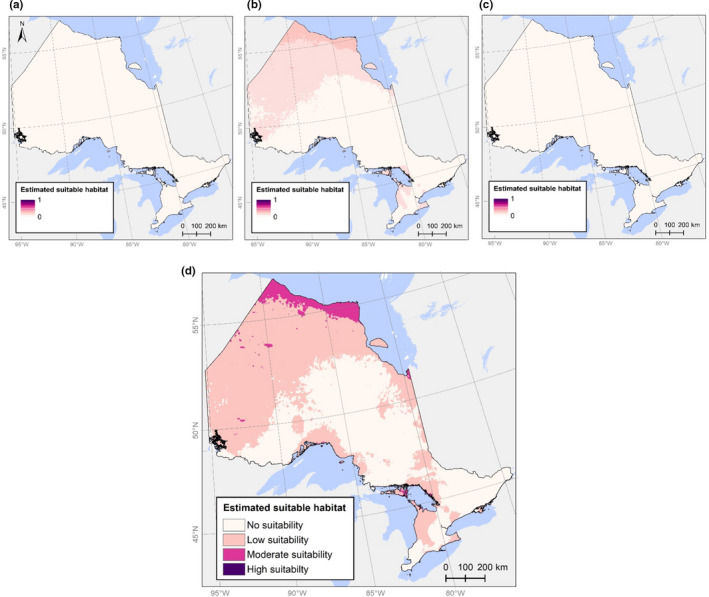
Reduced Model output of forecasted habitat suitability for *Bsal* using the ClogLog distribution using all available occurrence data for *Bsal* from (a) Asia and Europe, (b) Asia only, (c) Europe only. (d) Classification of *Bsal* habitat suitability projection based on the model using only Asia and all 29 variables. (<0.25 = no suitability, 0.25–0.5 = low suitability, 0.5–0.75 = moderate suitability, >0.75 = high suitability), based on the methods of Basanta et al. (2019). These results are unlikely to be accurate or informative for making meaningful predictions about *Bsal* habitat suitability in our study area. We therefore excluded these results from further analyses or interpretation in the main text. However, we have chosen to include them in this Appendix because they highlight the challenges in extrapolating Species Distribution Models among regions with vastly different climates and conditions

Our *Bsal* SDMs all predicted low habitat suitability across our study area, based on extrapolation from *Bsal* occurrences in Asia and Europe. They predicted very little suitable habitat in southern Ontario, and the highest suitability fell along the coast of Hudson's Bay, at the northern limit of the province. This result was clearly not biologically meaningful and is most likely caused by our attempt to predict future distributions based on data from regions with very different climatic conditions. We therefore discarded our predictive *Bsal* SDMs and did not pursue this approach further, although the results are presented in the Appendix A (Figure A7) for transparency.

#### 3.3.2 Predicted high‐risk areas for *Batrachochytrium salamandrivorans* impacts

**FIGURE 4:**
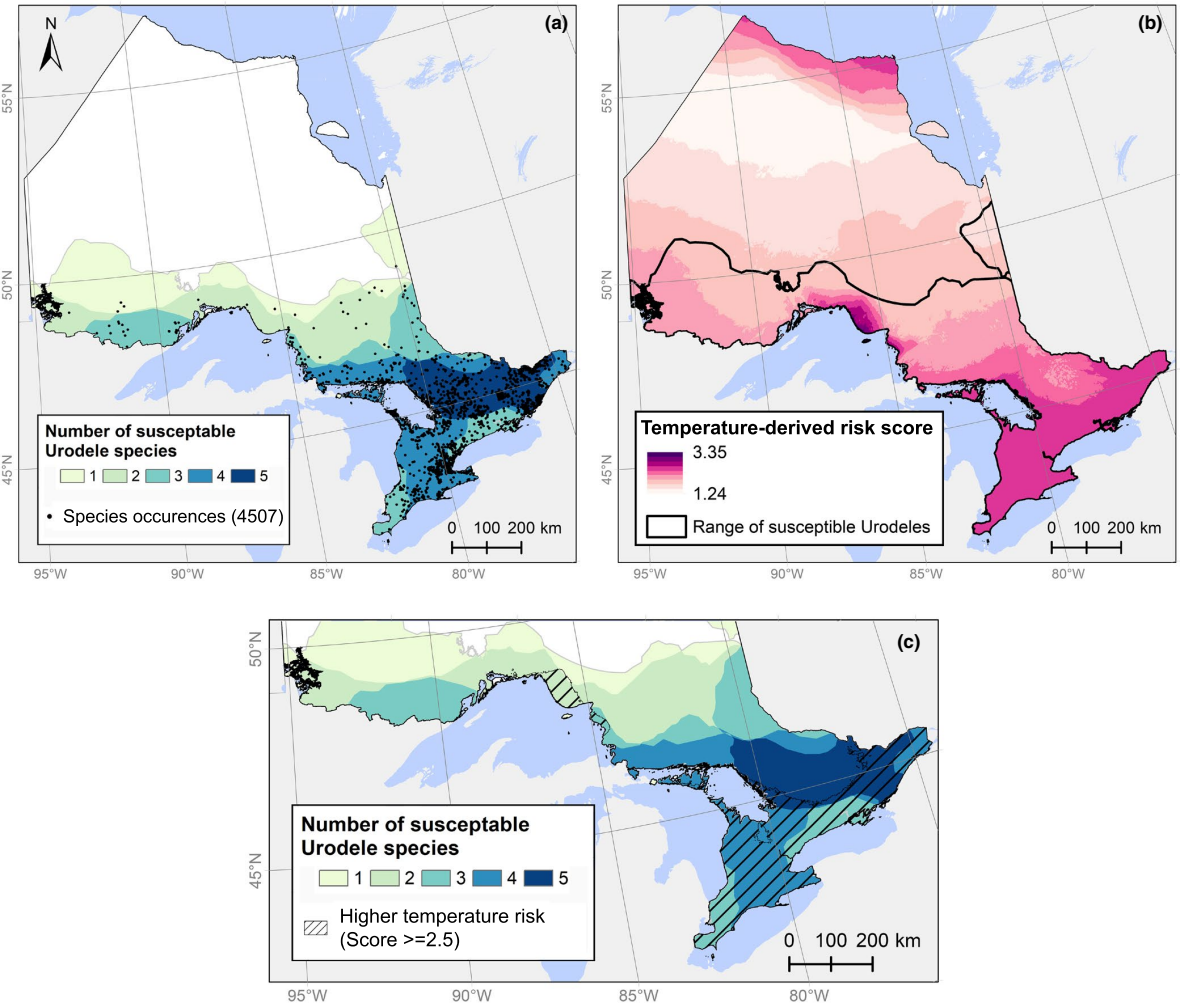
(a) Salamander richness map of five Urodele species likely susceptible to *Bsal*. All point data were collected between 1950 and 2020 and are a combination of our records and GBIF occurrences (GBIF, 2020). (b) Temperature‐based *Bsal* risk scores following Carter et al. (2021). (c) Area of overlap between high species richness (>4 species) and higher temperature risk scores (>=2.5), which covers 60,530 km^2^

As informative SDMs for our study area could not be produced with the available data, we instead used temperature as a proxy for likely severity of *Bsal* impacts if the pathogen were introduced, following Carter et al. (2021). Our species richness map (Figure 4a) indicated that 13% of the study area (119,969 km^2^) is home to ≥4 vulnerable species of salamander. Temperature‐derived risk scores ranged from 1.24 to 3.35 across our study area. The highest predicted risk scores were assigned to an area on the north shore of Lake Superior, followed by all of southern Ontario, and part of the coast of James Bay (Figure 4b). Over 90,123 km^2^, or 9.8% of the province, had scores ≥2.5 indicating moderate to high vulnerability (Figure 4c). By overlaying areas with ≥4 vulnerable salamander species and areas with temperature‐derived risk scores ≥2.5, we identified high‐risk areas covering 60,530 km^2^ (6.6% of Ontario) that could be prioritized for *Bsal* surveillance or mitigation if an introduction occurs (Figure 4c). The two layers overlapped substantially; 50.4% of the area containing ≥4 vulnerable species also fell within the higher temperature vulnerability zone.

## 4 DISCUSSION

We used opportunistic sampling to conduct surveillance for two species of chytrid fungus across Ontario and Akimiski Island and used positive *Bd* cases to model the niche of *Bd* in the study area. We detected *Bd* at all sites with ≥18 samples, and in all species with >25 samples. Only one detection was associated with potential clinical signs of chytridiomycosis. We detected *Bd* from the southernmost part of Ontario, to the Hudson Bay Lowlands in the north, and we found no evidence that *Bd* prevalence varied with latitude. Our results suggest that *Bd* is ubiquitous and enzootic across our study area (See also D’Aoust‐Messier et al., 2015; Fisher & Garner, 2020; Ouellet et al., 2005). We were encouraged that we did not detect *Bsal* in our sampling. Although our *Bsal* SDMs proved uninformative, they nicely illustrate the risks (as outlined in the Introduction) of constructing predictive SDMs that rely on occurrence records from very different climatic regions. In lieu of SDMs, we were able to more coarsely identify high‐risk areas for *Bsal* invasion by overlaying temperature‐derived risk scores with range maps of vulnerable species. These results can inform *Bsal* surveillance in Ontario, and once *Bsal* occurrence data from more representative locations become available, it will be possible to generate more informative, predictive SDMs.

Our estimates of *Bd* prevalence support previous surveillance work in eastern North America showing persistent occurrence of *Bd*, with samples collected across Canada (British Colombia, southern Ontario, and Atlantic Canada) detecting *Bd* prevalence ranging from 13.1–100%, (Forzán et al., 2010; Longcore et al., 2007; McMillan et al., 2020; Ouellet et al., 2005). In our study, the estimated prevalence of *Bd* should be interpreted as estimates of minimum prevalence. Surveillance based on qPCR can only detect fungal loads above the detection threshold, missing cases where pathogen load is low. We also used skin swabs that are non‐invasive and easy to replicate, but are less sensitive for detecting *Batrachochytrium* than tissue samples (Schock et al., 2010). These factors all lead to underestimates of true prevalence. Likewise, the results of our SDMs represent minimum estimates of suitable habitat for *Bd*, because we used a relatively small number of presence points (*N* = 86; Proosdij et al., 2016). Some areas were also undersampled as they do not have road access and are more difficult to reach. Nondetection of *Bd* from areas such as the Hudson Bay coast probably reflect low power to detect the pathogen with small sample sizes, rather than lower prevalence in the north. Despite these limitations, we detected *Bd* as far north as Akimiski Island, which had an average temperature of −1.25°C over our study period, and our most conservative SDM for *Bd* predicted that suitable habitat extends into the sub‐arctic (54°38′N). *Bd* grows slowly or not at all at temperatures <4°C, but can overwinter at lower temperatures (Piotrowski et al., 2004). Zoospores have slower rates of growth at lower temperatures, but exhibit longer activity, which may increase encounter rates with amphibian hosts (Voyles et al., 2012; Woodhams et al., 2008). Thus, the thermal sensitivity of *Bd* allows it to tolerate conditions common to our study area, contrary to previous niche models that identified the boreal and northern parts of North America as unsuitable habitat (Rödder et al., 2010; Xie et al., 2016). Including regionally relevant occurrence data in our *Bd* SDMs resulted in a much broader predicted niche and highlighted the ubiquity of *Bd* in our sampling area.

Ironically, lack of regionally relevant occurrence data also prevented us from predicting suitable habitat for *Bsal*. We were still able to broadly identify areas where salamanders would be most at risk from a potential *Bsal* introduction. However, the maps we generated through this exercise represented a tool to highlight priority areas for surveillance in the event of a *Bsal* introduction, rather than a prediction of the full, available niche for *Bsal* in Ontario. Use of these maps to plan surveillance for *Bsal* must consider three important caveats. First, the species richness layers were created using broad‐scale range maps that did not capture local variation in the distribution of each species. Second, our risk scores for *Bsal* were derived from experimental infection of *N*. *viridescens* (Carter et al., 2021). Use of these risk scores required an explicit, untested assumption: that the effects of *Bsal* on *N*. *viridescens* can be generalized to other susceptible species. Recent work suggests that *N*. *viridescens* may be particularly vulnerable to the effects of *Bsal*, compared to cooccurring Plethodontids (DiRenzo et al., 2021). Our objective was to identify high‐risk areas based on current, available evidence—but these maps should be refined in the future when inter‐specific variation in susceptibility to *Bsal* and the thermal drivers of disease severity have been quantified. Third, we did not include *Ambystoma* species when summarizing salamander species richness. Genomic analyses suggest that *A*. *maculatum* is not vulnerable to *Bsal* (Martel et al., 2014). Skin peptides from *A*. *maculatum* inhibit the growth of *Bsal* (Pereira & Woodley, 2021), and experimentally infection with high, repeated doses of *Bsal* showed resistance across all life stages with no clinical signs of infection (Barnhart et al., 2020). These results suggest *Ambystoma* are not at risk from *Bsal* introduction, but future research should confirm whether *A*. *maculatum* accurately represents its congeners. Research is also needed to understand whether resistance to *Bsal* limits the potential for *Ambystoma* species to act as reservoir hosts for *Bsal*, or whether *Ambystoma* could carry and transmit *Bsal* to co‐occurring, susceptible species.

Although *Bd* was detected across our study area, chytridiomycosis was not—consistent with other studies from eastern North America showing persistent occurrence of *Bd* in the absence of disease. In southern Ontario, 28.9% of 2,223 *L*. *pipiens* tested positive for *Bd*, but no sick or dead specimens were observed (McMillan et al., 2020). On the east coast of Canada, *Bd* was detected in 26.9% of 115 sampled amphibians, but only a single, deceased *L*. *sylvaticus* exhibited clinical signs of disease (Forzán et al., 2010). Periods of high *Bd* prevalence in sampled roadkill were also not correlated with documented die‐offs in Maine, USA (Longcore et al., 2007). Chytridiomycosis severity varies with host susceptibility and strain virulence (Eskew et al., 2018; Gahl et al., 2012), and the strain or strains present in our study area may simply be less virulent than those occurring elsewhere. Continued surveillance can assess the potential emergence of more virulent strains, such as that observed in a population of Cascades frogs (*Rana cascadae*) in northern California (Piovia‐Scott et al., 2015). Nevertheless, although *Bd* has had severe impacts on many amphibian populations, amphibians in our study area appear to currently co‐exist alongside it with only intermittent outbreaks (Kilpatrick et al., 2010).

In contrast, the impacts of a *Bsal* introduction on salamander in our study area could be severe. *Bsal* grows optimally at 10–15°C, can survive between 5 and 25°C, and has a lower thermal preference than *Bd* (Martel et al., 2013). We identified southern Ontario and two regions farther north as likely high‐risk areas in the event of a *Bsal* introduction. Southern Ontario is one of the most densely populated regions in Canada, and wildlife in this area are already threatened by habitat loss and fragmentation (Paterson et al., 2021). The large cities in southern Ontario represent potential trade routes for introducing *Bsal* into the country (Stephen et al., 2015), highlighting international trade restrictions as an important policy tool (Auliya et al., 2016; Stegen et al., 2017). As of May 31, 2017, importation of salamanders into Canada was prohibited without a permit (Government of Canada, 2017). However, anurans can carry *Bsal* without exhibiting clinical signs of disease (Nguyen et al., 2017) and can transmit the pathogen to salamanders with fatal results (Stegen et al., 2017). Expanding trade restrictions to include anurans could further protect native amphibians.

In the era of broad‐scale ecological problems that require interdisciplinary solutions, incorporating team science and open science into data‐intensive ecological research can improve the scope and impact of research that can directly inform policy (Cheruvelil & Soranno, 2018). Our study represents a collaboration across research programs and agencies to reach shared goals of infectious disease surveillance. Opportunistic sampling required minimal equipment and expertise and not only provided an extensive dataset, but provided data from remote areas that would have been too costly to reach otherwise. This sampling enabled a more accurate estimate of the *Bd* niche near the northern range limits of its hosts, established a pre‐introduction baseline for future surveillance for *Bsal*, and identified priority areas for robust and sustained surveillance. In 2015, the Canadian Wildlife Health Cooperative released a report that recommended restricting the import of salamanders to Canada prior to the salamander ban (Stephen et al., 2015), and their recommendations remain relevant. These include not only amending import policies but also applying obligatory testing for *Bsal*, early detection and containment, and increasing awareness of the pathogen within the pet trade community. The current ban on salamander importation is encouraging, but the potential importation of *Bsal* on anuran hosts remains a concern. Policy reforms, continued surveillance, and international cooperation will be required to prevent potential introductions into North America and protect native amphibians.

## CONFLICT OF INTEREST

This study was funded by the Government of Ontario and the Canadian Wildlife Health Cooperative. The authors declare no conflicts of interest.

## AUTHOR CONTRIBUTIONS

**Lauren Crawshaw:** Formal analysis (lead); Visualization (lead); Writing – original draft (lead); Writing – review & editing (equal). **Tore Buchanan:** Conceptualization (equal); Data curation (lead); Project administration (supporting); Resources (equal); Writing – review & editing (equal). **Leonard Shirose:** Conceptualization (lead); Methodology (supporting); Project administration (supporting); Resources (equal); Writing – review & editing (equal). **Amanda Palahnuk:** Data curation (equal); Investigation (equal); Writing – review & editing (equal). **Hugh Y. Cai:** Investigation (equal); Writing – review & editing (equal). **Amanda M. Bennett:** Formal analysis (supporting); Writing – review & editing (equal). **Claire M. Jardine:** Conceptualization (equal); Project administration (supporting); Resources (equal); Writing – review & editing (equal). **Christina M. Davy:** Conceptualization (equal); Data curation (lead); Formal analysis (supporting); Investigation (lead); Methodology (lead); Project administration (lead); Supervision (lead); Writing – original draft (lead); Writing – review & editing (equal).

### OPEN RESEARCH BADGES

This article has earned an Open Data Badge for making publicly available the digitally‐shareable data necessary to reproduce the reported results. The data is available at https://doi.org/10.17605/OSF.IO/7XNRE.

## Data Availability

All data and analytical code used for this study are archived as an OSF project at https://osf.io/7xnre/. https://doi.org/10.17605/OSF.IO/7XNRE.

## APPENDIX A

**TABLE A1:** Correlation Matrix of 19 bioclimatic variables and Digital Elevation Model (DEM) used to reduce the number of variables in the *Bd* models

	BIO1	BIO2	BIO3	BIO4	BIO5	BIO6	BIO7	BIO8	BIO9	BIO10	BIO11	BIO12	BIO13	BIO14	BIO15	BIO16	BIO17	BIO18	BIO19	DEM
BIO1	1																			
BIO2	−0.13	1																		
BIO3	0.72	0.49																		
BIO4	**−0.92**	0.13	**−0.79**	1																
BIO5	**0.86**	0.1	0.59	−0.61	1															
BIO6	**0.97**	−0.27	0.68	**−0.95**	**0.75**	1														
BIO7	**−0.88**	0.37	−0.62	**0.96**	−0.54	**−0.96**	1													
BIO8	0.14	−0.15	−0.19	0.12	0.36	0.06	0.07	1												
BIO9	**0.9**	−0.21	0.69	**−0.92**	0.66	**0.94**	**−0.92**	−0.07	1											
BIO10	**0.94**	−0.18	0.53	−0.73	**0.95**	**0.87**	−0.72	0.35	**0.78**	1										
BIO11	**0.99**	−0.14	**0.75**	**−0.96**	**0.79**	**0.99**	**−0.93**	0.05	**0.93**	**0.89**	1									
BIO12	0.68	0.26	**0.84**	**−0.81**	0.43	0.67	−0.67	−0.33	0.7	0.45	0.73	1								
BIO13	0.45	0.13	0.54	−0.55	0.26	0.46	−0.47	−0.18	0.45	0.27	0.48	0.73	1							
BIO14	0.74	0.12	**0.77**	**−0.84**	0.5	0.73	−0.72	−0.28	**0.76**	0.55	**0.79**	**0.9**	0.54	1						
BIO15	−0.62	−0.3	**−0.76**	0.7	−0.46	−0.58	0.55	0.25	−0.62	−0.46	−0.66	**−0.77**	−0.2	**−0.84**	1					
BIO16	0.58	0.21	0.69	−0.68	0.38	0.57	−0.56	−0.24	0.58	0.39	0.62	**0.9**	**0.9**	0.73	−0.46	1				
BIO17	**0.76**	0.16	**0.83**	**−0.87**	0.5	**0.75**	**−0.75**	−0.32	**0.8**	0.54	**0.81**	**0.94**	0.55	**0.97**	**−0.87**	**0.76**	1			
BIO18	0.48	0.3	0.58	−0.49	0.41	0.41	−0.36	0.08	0.39	0.38	0.48	0.74	0.71	0.6	−0.4	**0.82**	0.6	1		
BIO19	0.61	0.18	**0.79**	**−0.79**	0.3	0.64	−0.69	−0.48	0.73	0.35	0.68	**0.93**	0.58	**0.86**	**−0.8**	**0.75**	**0.93**	0.54	1	
DEM	0.38	0.06	0.22	−0.32	0.4	0.28	−0.19	0.08	0.27	0.38	0.36	0.42	0.35	0.53	−0.3	0.48	0.42	0.54	0.21	1

Data are derived from the Daily Surface Weather Data Version 3 (Daymet) and the Shuttle Radar Topography Mission (Farr et al., 2007; NASA JPL, 2013). Variables with a correlation *R* > 0.75 are presented in bold.

**TABLE A2:** Species in dataset obtained from our occurrence records and the Global Biodiversity Information Facility (GBIF, 2020)

Species	Common name	*N* (%)
Plethodontidae		
*Plethodon cinereus*	Eastern red‐backed salamander	2,843 (63.1)
*Eurycea bislineata*	Two‐lined salamander	193 (4.3)
*Hemidactylium scutatum*	Four‐toed salamander	117 (2.6)
Proteidae		
*Necturus maculosus*	Common mudpuppy	234 (5.2)
Salamandridae		
*Notophthalmus viridescens*	Eastern newt	1,120 (24.9)
	TOTAL	4507

These species are confirmed or considered potentially susceptible to *Bsal* (Martel et al., 2014).

**TABLE A3:** Percent contribution and permutation importance of each environmental predictor of the model with the best performance (Best model: Bias Grid 0.5, Reduced variables)

Variable	Percent contribution	Permutation importance
Min Temperature of Coldest Month (BIO6)	41.2	12.7
Mean Diurnal Range (BIO2)	16.7	7.2
Isothermality (BIO3)	10.6	2.9
Precipitation of Warmest Quarter (BIO18)	6.8	14.8
Max Temperature of Warmest Month (BIO5)	6.6	9.3
Precipitation of Wettest Quarter (BIO16)	6.4	33.7
Elevation (m)	4.4	3.1
Mean Temperature of Wettest Quarter (BIO8)	4.3	10.4
Precipitation Seasonality (BIO15)	2.9	5.8
